# Uganda Genome Resource: A rich research database for genomic studies of communicable and non-communicable diseases in Africa

**DOI:** 10.1016/j.xgen.2022.100209

**Published:** 2022-11-09

**Authors:** Segun Fatumo, Joseph Mugisha, Opeyemi S. Soremekun, Allan Kalungi, Richard Mayanja, Christopher Kintu, Ronald Makanga, Ayoub Kakande, Andrew Abaasa, Gershim Asiki, Robert Kalyesubula, Robert Newton, Moffat Nyirenda, Manj S. Sandhu, Pontiano Kaleebu

**Affiliations:** 1 The African Computational Genomics (TACG) Research Group, MRC/UVRI and LSHTM, Entebbe, Uganda; 2 The Department of Non-communicable Disease Epidemiology, London School of Hygiene and Tropical Medicine London, London, UK; 3 Medical Research Council/ Uganda Virus Research Institute/London School of Hygiene and Tropical Medicine (MRC/UVRI/LSHTM) Uganda Research Unit, Entebbe, Uganda; 4 College of Health Sciences, Makerere University, Kampala, Uganda; 5 Health and Systems for Health Research Unit, African Population and Health Research Center, Nairobi, Kenya; 6 Department of Epidemiology and Biostatistics, School of Public Health, Imperial College London, London, UK

**Keywords:** genomics, Uganda, non-communicable diseases, NCDs, general population cohort, GPC

## Abstract

The Uganda Genome Resource (UGR) is a well-characterized genomic database with a range of phenotypic communicable and non-communicable diseases and risk factors generated from the Uganda General Population Cohort (GPC), a population-based open cohort established in 1989. The UGR comprises genotype data on ∼5,000 and whole-genome sequence data on ∼2,000 Ugandan GPC individuals from 10 ethno-linguistic groups. Leveraging other platforms at MRC/UVRI and LSHTM Uganda Research Unit, there is opportunity for additional sample collection to expand the UGR to advance scientific discoveries. Here, we describe UGR and highlight how it is providing opportunities for discovery of novel disease susceptibility genetic loci, refining association signals at new and existing loci, developing and testing polygenic scores to determine disease risk, assessing causal relations in diseases, and developing capacity for genomics research in Africa. The UGR has the potential to develop to a comparable level of European and Asian large-scale genomic initiatives.

## Graphical abstract

**Figure:**
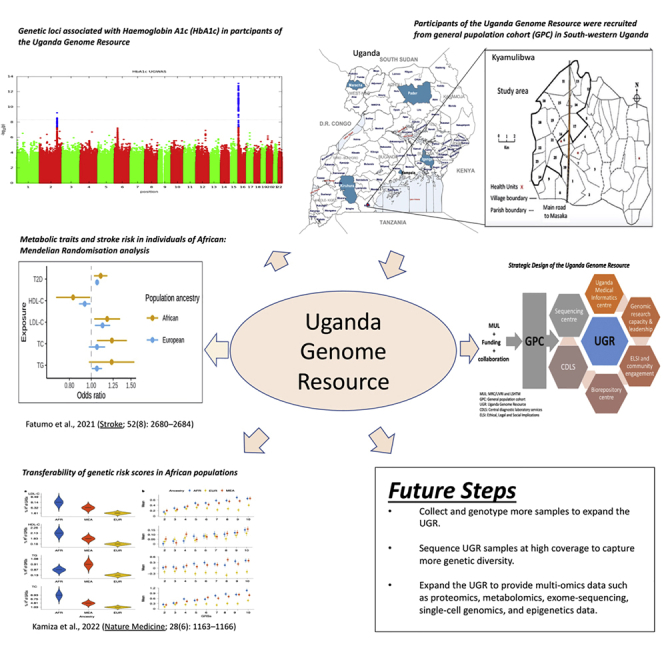

## Teaser

Fatumo et al. describe how a well-characterized genotype and phenotype dataset (Uganda Genome Resource, UGR) has so far provided insights into the genetic nature of several traits and diseases. They further describe opportunities the UGR presents for collaborative studies and how it could be expanded to advance scientific discoveries.

## Introduction

The genetic diversity in Africa is far greater than in any other region across the globe, but, unfortunately, the vast majority of genomic studies have been performed in European ancestry populations.^1^^,^^2^ Uganda is located in East Africa with four major ethnic groups and over 40 languages. The rich linguistic, ethnic, and cultural diversity of Uganda provides an unprecedented opportunity to understand the level of the genetic structure in Ugandan populations. To advance genetic epidemiology of communicable and non-communicable diseases (NCDs) in Uganda, the Uganda Genome Resource (UGR) was launched in 2011 by the Medical Research Council (MRC)/Uganda Virus Research Institute (UVRI) and LSHTM Uganda Research Unit (https://www.lshtm.ac.uk/research/units/mrc-uganda) in collaboration with Wellcome Sanger Institute and the University of Cambridge to prospectively collect a wide range of NCDs; infectious disease risk factors including information on lifestyle, family history social determinant, demographics, sexual health and reproductive behavior, past illness, mental health, treatment and immunization; and environmental risk factors.^3^ Currently, a study is being undertaken in the general population cohort (GPC) to study the genetic and environmental risk factors for diabetes and hypertension.

Here, we provide a detailed description of the UGR, which is different from previous publications on the GPC that focused on specific aspects^3^ or population genetics and genome-wide association analyses of cardiometabolic traits in UGR data.^4^ We aim to give an overview of UGR as a resource including detailed phenotype availability, genomic data generation, sample characteristics, genetic discoveries to date, and, finally, its data access and sharing policy.

## Study population: the general population cohort

The GPC is a population-based study of approximately 22,000 individuals residing in 25 neighboring villages in the Kyamulibwa sub-county, Kalungu district in rural southwestern Uganda. The study was founded in 1989 by the Medical Research Council UK (MRC UK) in collaboration with the UVRI to study the epidemiology of HIV in a general population. The GPC was initially recruited and assessed through annual house-to-house census and survey rounds until 2012, when biannual surveys commenced. Since its establishment, 26 rounds of survey and 29 rounds of census have been undertaken. Before any survey procedures are carried out, written informed consent is obtained from participants on the use of their clinical records for research purposes and sample storage for future use.^3^ Data collected includes serological, demographic, and medical information from participants. Information regarding mortality, fertility, sexual behavior migration, and HIV infection perception are routinely collated.

**Figure 1:**
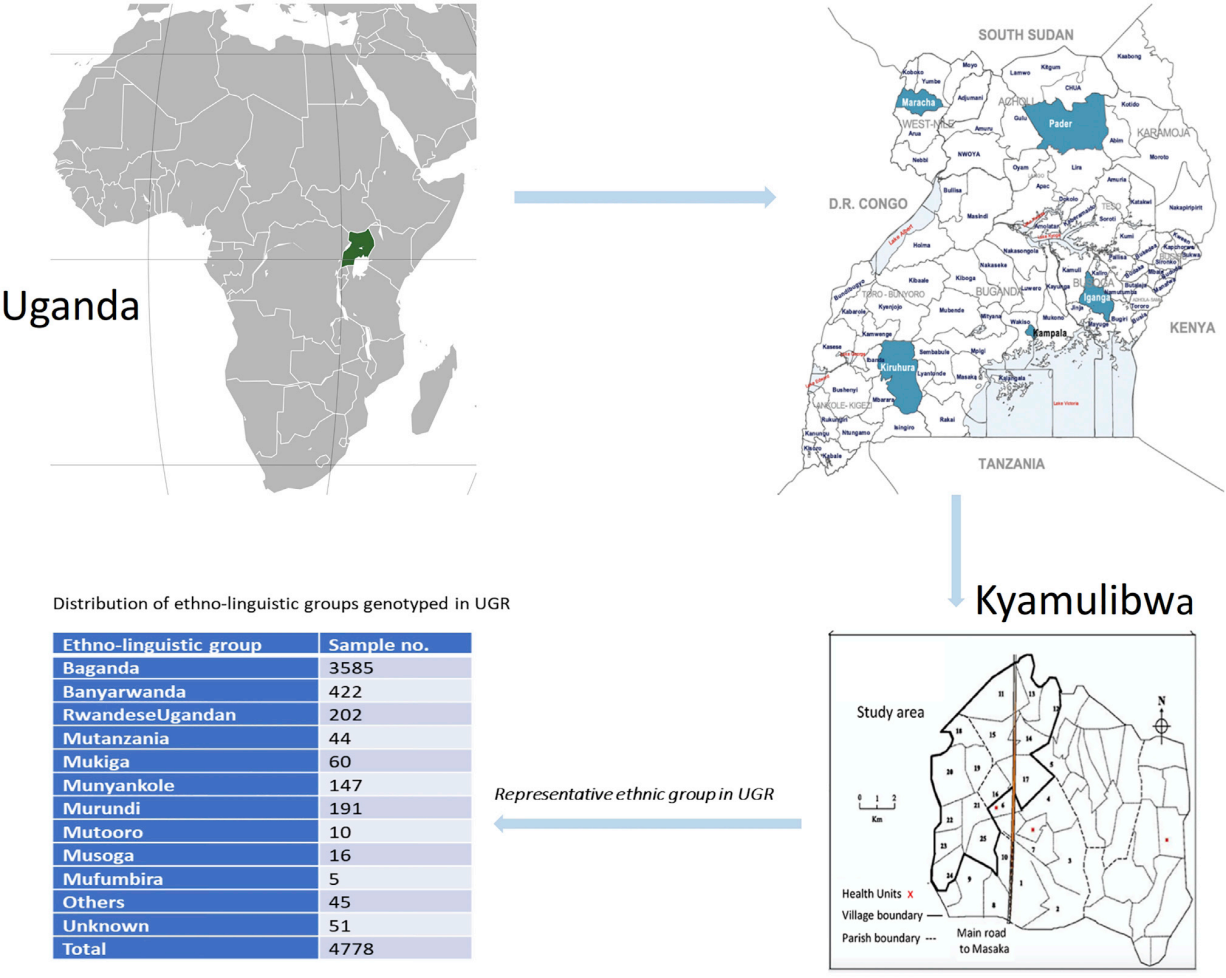
Distribution of the samples with genotyped data in the Uganda Genome Resource

The GPC round 22 study of 2011 focused on the genetics and epidemiology of communicable disease and NCD, capturing different ethnic groups in Uganda for genomic studies (Figure 1). The survey round that was used to establish the UGR consisted of five main stages, including mobilization (recruitment and consenting), mapping, census, survey, and results feedback and clinical follow-up. The specific objectives of this survey then were:

1. To create a one-of-a-kind study for expanding on a large-scale prospective cohort research in an African population to evaluate a wide range of health indices and to lay the platform for longer-term investigations.
2. To provide etiological insights into variance in cardio-metabolic and infectious risk factors using population, genetic, and epidemiological techniques.
3. To help develop public health policies in other African countries by informing health policy and public health programs aimed at addressing the rise in NCDs in Uganda.

The cohort continues to function as a valuable platform for investigating the relationship between communicable illnesses and NCDs in a regular annual survey of the GPC.

**Figure 2:**
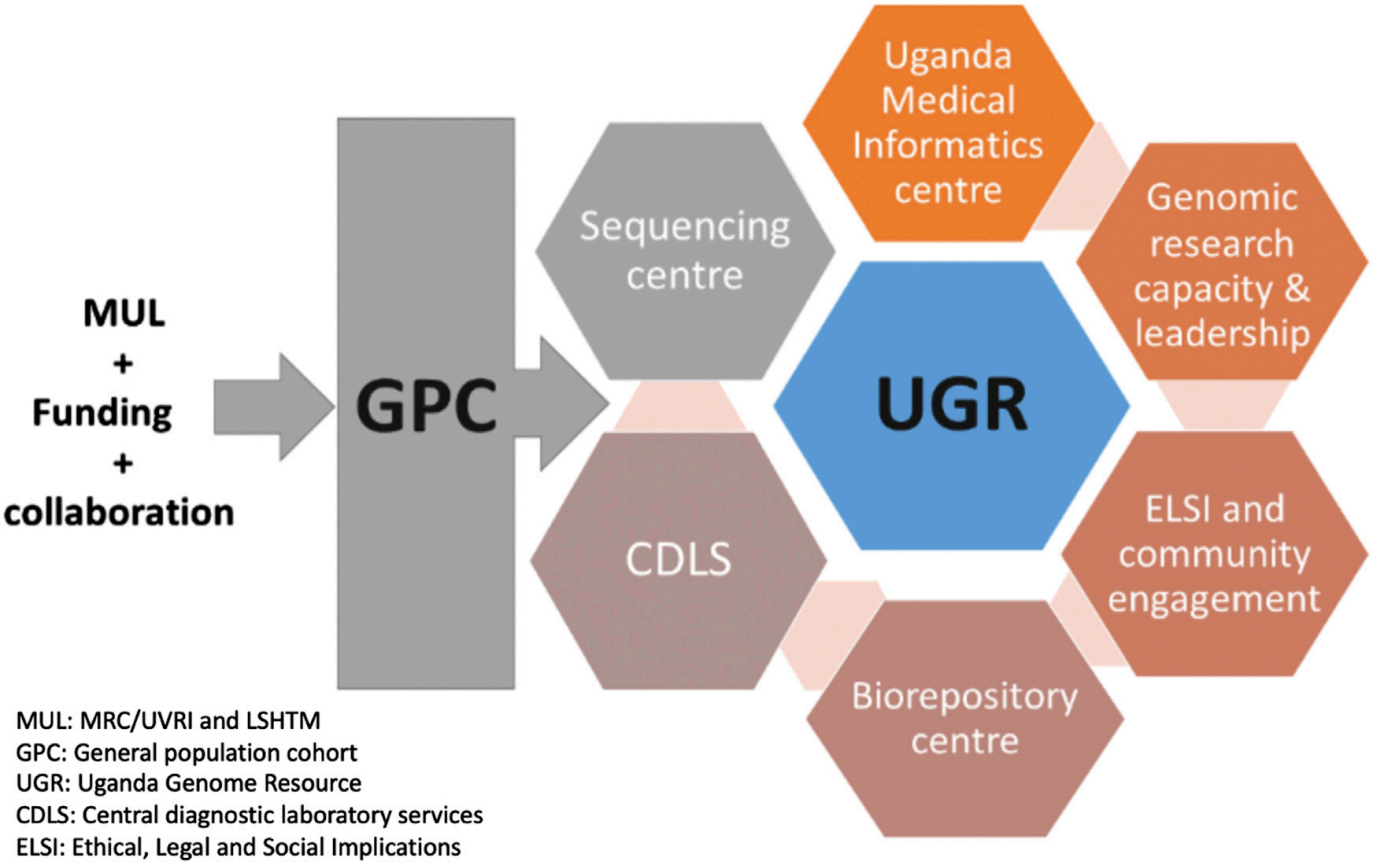
Strategic design of the different platforms which support the Uganda Genome Resource

As shown in Figure 2, the UGR is supported by different platforms at the MRC/UVRI and LSHTM Uganda Research Unit (the Unit). The Unit has a reputation for leadership in genomics research capacity. The clinical diagnostic laboratory (CDLS) is an ISO 15189-certified laboratory that provides high-quality diagnostics testing support at the Unit (https://www.lshtm.ac.uk/research/units/mrc-uganda/clinical-diagnostic-laboratory-services). The Uganda Medical Informatics Centre (UMIC) is currently one of the largest health research-orientated computational resources in Sub-Saharan Africa with modern high-performance computing facilities (https://www.lshtm.ac.uk/research/units/mrc-uganda/bioinformatics-section) to collect, store, and analyze data to advance genomic research. The Unit is also supported by a well-organized community engagement structure and activities, with strong support from the Community Advisory Board (CAB) including a biorepository for biospecimen storage and a DNA sequencing center.

## Community engagement and the consenting process

The Unit has a community advisory group for the GPC for which most members are leaders from the community. The Unit has a dedicated communication office that helps in public engagement, including the dissemination of research findings and coordinating the engagement exercise. For GPC round 22 in which genetic samples for the UGR data were collected, meetings were held with the GPC CAB, which comprised representatives from different constituencies. In the meetings, the CAB activities and research procedures that were to be carried out were discussed.

The CAB meetings were followed by community meetings that were held within each study village prior to the commencement of the GPC round. These meetings were between the research team and community members. At these meetings, the details concerning the survey round were discussed. For any issues raised regarding the study, the research team and the community discussed ways to solve them.

For the surveys, participants were mobilized to come to the survey hubs on specific days. On the day of the survey, an information sheet with details about the study was read to the study participant. If the participant had any questions, these were answered by the research staff administering the information sheet. If the participant agreed to participate in the survey, they then signed or provided their thumbprint on the consent form.

## Ethics

The study was approved by the Science and Ethics Committee of the UVRI Research and Ethics Committee (UVRI-REC #HS 1978), the Uganda National Council for Science and Technology (UNCST #SS 4283), and the East of England-Cambridge South (formerly Cambridgeshire 4) NHS Research Ethics Committee UK.

## Genotype generation, quality control, and imputation

The 2.5M Illumina chip array was used to genotype nearly 5,000 Ugandans at the Wellcome Trust Sanger Institute, and quality control steps have been presented.^4^ In summary, we used a strict quality control process to perform a series of steps in a logical order to eliminate a total of 39,368 autosomal markers that failed to meet the quality metrics for single-nucleotide polymorphism (SNP) call rate (>97%, 25,037 SNPs) and Hardy-Weinberg equilibrium (HWE) (p < 1 × 10^−8^, 14,331 SNPs). During sample quality control, a total of 91 samples were eliminated because they failed the quality standards for sample call rate (>97%) or heterozygosity (observed heterozygosity HO = 0.209333 ± 0.007416 matching to the mean ± 3 SD) or the sex extrapolated from the X chromosome did not correspond to the reported sex. Three further samples were eliminated because of high relatedness (identical by descent, IBD > 0.90). There were no samples that were classified as outliers in terms of population or ancestry. A total of 2,230,258 autosomal markers and 4,778 samples (Figure 1) that met the stated threshold were subjected to further analysis. We carried out SNP phasing with the aid of SHAPEIT2^5^ using default settings, and then imputation was done with IMPUTE2.^6^ All samples were imputed with a combined reference that was created by combining the UG2G sequence resource (n = 2,000, whole-genome sequence data from the African Genome Variation Project [n = 320]) and the 1000 Genomes phase 3 project (n = 2,504). The principal-component analysis (PCA) plot for the GPC participants (n = 4,778) was published.^4^

All participants for the UGR were recruited from the same geographical region in the 25 villages. Figure 1 shows the geographical location of Kyamulibwa, the sub-county from where UGR participants were recruited. Uganda is home to several diverse ethno-linguistic groups, the most common being Baganda found in Central Uganda. A total of 10 ethno-linguistic groups were reported in the UGR (Figure 1), the majority being Baganda, comprising 75% of the entire UGR participants. The population structure among these 10 ethno-linguistic groups has been reported elsewhere.^4^

## Whole-genome sequence data (Uganda 2000 genomes; UG2G)

The entire genomes of more than 2,000 Ugandans from 10 ethno-linguistic groups were sequenced using the Illumina HiSeq 2000 with 75 bp paired-end reads at low coverage, with an average coverage of 4× for each sample. 343 of these samples overlapped with people who had already been genotyped. An automated quality control process was used to bring down the data files that needed manual processing to ascertain the quality of binary alignment map (BAM) files produced. This method was based on the one developed for the UK10K project,^7^ which used a set of algorithmically derived standards to determine summary data computed from the input BAMs. Any line that fell below the “fail” standard for any of the metrics was deleted; lines falling below the “warn” standard for any of the scores were manually investigated; and any line that passed any of these scores was given a status of “pass.” Overall, we deleted 14 samples from the study. Full details on the quality control and how we computed the summary data have been described by Gurdasani and his colleagues.^4^

## Merging of sequenced and genotyped data

We integrated sequenced and imputed genotyped data to produce an aggregated dataset to boost power for discovery in genome-wide association studies (GWASs). The call rates for the merged sequenced and genotyped data were not affected despite the low coverage (4×) for the sequenced data.^4^ Because cryptic and family relatedness persisted across sequenced and genotyped data, we produced an aggregated dataset for analysis instead of separately meta-analyzing the data, because data would be correlated rather than independent. As a result, conclusions from mixed-model analysis that explicitly model this relationship are more likely to be true. We examined and deleted any consistent discrepancies between sequences and imputed genotype data after merging the two datasets. This was done by performing PCA on the dataset to see whether there was any distinction by data modality (imputed genotype data versus sequenced data) among the 343 people who had their genotypes and sequences done in duplicate. On PCA, we noticed a strong separation of genotype imputed and sequence data points. For these 343 samples, we tested alternative concordance criteria between sequencing and imputed genotype data, screening out SNPs with a concordance of 0.80 and 0.90 in the dataset. In the UGR, we discovered that to eliminate systematic effects detected between genotyping array and sequence data on PCA, a minimum concordance criterion of 0.90 was necessary.

There were no systematic changes between sequenced and genotyped data in PCAs after excluding 904,283 SNPs that exhibited 90% concordance in genotypes between the sequence and imputed genotype data. We examined the top 10 principal components to confirm that systematic variations in the genomic data did not constitute an important axis of variation. After filtering, a total of 39,312,112 autosomal markers were taken forward for analysis in a joint dataset of 6,407 samples (please see Gurdasni et al.^4^ for details).

## Phenotype data and laboratory measurement

During survey round 22, which was conducted in 2011, several phenotypes based on clinical and physical examinations, laboratory tests, and self-reported questionnaires were collected from the respondents (Table 1), and these respondents who are still known to be alive and have not moved out of the GPC have been followed every year since then. A blood specimen was analyzed for non-fasting blood lipids, blood cell traits (mean cell hemoglobin, red cell count, white cell count, mean cell hemoglobin concentration, hemoglobin, packed cell volume, mean cell volume, and platelet), glycemic characteristics, renal function, and infectious biomarkers (HIV, hepatitis B and C). Basic demographics data such as age, sex, marital status, and education level are available (Table 1). Data on anthropometrics such as BMI, weight, waist-to-hip ratio, and height; blood pressure measurements; as well as lifestyle information such as smoking status, physical activities, and diet were also collected (Table 1). Data are also available on sexual health and reproductive behavior, sex education, condom use, pregnancy and outcome, and number of offspring (Table 1). Leveraging the biorepository and CDLS platforms at the Unit, we tested stored biosamples for new phenotypes, e.g., serum creatinine, albuminuria, and blood urea nitrogen, to expand our studies on the genetics of kidney function and a new phenotype that allows for global collaboration such as the Global Biobank Meta-analysis Initiative (GBMI).^8^ We are also expanding data collection within GPC to include respiratory function and mental health phenotypes, such as major depressive disorder and schizophrenia.

**Table 1:** Sample characteristics of participants of the Uganda Genome Resource at baseline

Characteristic	All (n = 7,833)	Males (n = 3,425)	Female (n = 4,404)
Number of individuals interviewed, N	7,833	3,425	4,404
Age (years), median (IQR)	30 (17–46)	27 (17–44)	31 (19–47)

Age group (years), N (%)			

13–19	2,398 (30.6)	1,218 (35.6)	1,180 (26.8)
20–29	1,475 (18.8)	612 (17.9)	863 (19.6)
30–39	1,311 (16.8)	495 (14.5)	816 (18.5)
40–49	1,047 (13.4)	439 (12.8)	608 (13.8)
50–59	711 (9.1)	297 (8.7)	414 (9.4)
60+	887 (11.3)	364 (10.6)	523 (11.9)
Body mass index (kg/m2), mean ± SD	21.2 ± 3.83	20.1 ± 3.11	22.0 ± 4.13

BMI classification, N (%)			

Underweight	1,712 (22.6)	1,031 (30.4)	681 (16.3)
Normal	4,919 (65.0)	2,188 (64.4)	2,731 (65.4)
Overweight	739 (9.8)	156 (4.6)	583 (14.0)
Obese	201 (2.6)	21 (0.6)	180 (4.3)

Smoking status, N (%)			

Current smoker	641 (8.2)	553 (16.2)	88 (2.0)
Ex-smoker	194 (2.5)	169 (4.9)	25 (0.6)
Never smoked	6,990 (89.3)	2,700 (78.9)	4,290 (97.4)

Alcohol consumption, N (%)			

Never or no alcohol use	5,040 (70.1)	2,052 (64.6)	2,988 (74.4)
Infrequent current drinker	537 (7.5)	165 (5.2)	372 (9.3)
Frequent current drinker	1,618 (22.5)	961 (30.2)	657 (16.4)

Cardio metabolic quantitative measurements (mean ± SD)			

Total cholesterol (mmol/L), mean ± SD	3.5 ± 0.98	3.3 ± 0.91	3.7 ± 1.00
High-density lipoprotein (mmol/L), mean ± SD	1.0 ± 0.41	0.9 ± 0.42	1.0 ± 0.40
Low-density lipoprotein (mmol/L), mean ± SD	2.0 ± 0.77	1.8 ± 0.63	2.1 ± 0.80
Albumin, mean ± SD	41.3 ± 4.10	41.8 ± 4.15	40.9 ± 4.02
HbA1c, mean ± SD	3.3 ± 0.68	3.3 ± 0.69	3.3 ± 0.73
Triglycerides (mmol/L), mean ± SD	1.2 ± 0.61	1.1 ± 0.61	1.2 ± 0.62
Alanine aminotransferase, median (IQR)	14.0 (17.8–22.9)	19.4 (15.6–25.1)	13.0 (16.4–21.3)
Alkaline phosphatase, median (IQR)	71.3 (92.5–144.1)	74.3 (96.8–208.0)	68.5 (89.5–123.1)
Aspartate aminotransferase, median (IQR)	21.2 (25.1–30.4)	23.8 (28.0–33.0)	19.8 (23.1–27.4)
Gama glutamyltransferase, median (IQR)	13.5 (18.7–28.0)	15.6 (21.6–33.0)	12.2 (17.0–24.2)
Bilirubin, median (IQR)	5.2 (7.7–12.0)	5.92 (8.9–14.2)	4.8 (6.9.0–10.5)

Anthropometric Measurements			

Weight (kg), mean ± SD	52.6 ± 11.35	52.4 ± 11.37	52.7 ± 11.33
Height (cm), mean ± SD	157.2 ± 9.19	160.7 ± 10.54	154.5 ± 6.83
Systolic blood pressure (mmHg), mean ± SD	122.4 ± 17.0	123.5 ± 16.2	121.6 ± 17.51
Diastolic blood pressure (mmHg), mean ± SD	74.2 ± 10.26	73.5 ± 10.39	74.7 ± 10.12

Anemia			

White blood cell count	5.1 ± 1.51	5.1 ± 1.58	5.1 ± 1.58
Red blood cell count	4.7 ± 0.62	4.9 ± 0.65	4.6 ± 0.56
Hemoglobin	13.6 ± 1.62	14.2 ± 1.74	13.1 ± 1.33
Waist-hip ratio	0.85 ± 0.16	0.86 ± 0.17	0.8 ± 0.16
Mean corpuscular hemoglobin	28.9 ± 2.91	29.1 ± 2.92	28.8 ± 2.90
Mean corpuscular hemoglobin concentration;	33.7 ± 1.19	33.7 ± 1.24	33.7 ± 1.15
Red blood cell distribution width	13.1 ± 1.34	13.1 ± 1.40	13.1 ± 1.29
Mean platelet volume	8.7 ± 0.83	8.7 ± 0.83	8.7 ± 0.82
Platelet count (PLT)	216.9 ± 77.7	207.9 ± 77.3	223.9 ± 77.30
Lymphocytes	2.4 ± 0.83	2.5 ± 0.92	2.4 ± 0.76
Monocytes	0.3 ± 0.12	0.29 ± 0.14	0.3 ± 0.11
Basophils	0.05 ± 0.04	0.05 ± 0.42	0.05 ± 1.58
Neutrophils	1.9 ± 0.86	1.9 ± 0.84	2.0 ± 0.88
Eosinophils	0.35 ± 0.39	0.4 ± 0.40	0.3 ± 0.39

Vaccination			

Received BCG vaccine, N (%)			

Yes	1,421 (18.2)	631 (18.4)	790 (18.0)
No	553 (7.1)	221 (6.5)	332 (7.5)

Don’t know	5,850 (74.8)	2,570 (75.1)	3,280 (74.5)

Received oral polio vaccine, N (%)			

Yes	1,398 (17.9)	612 (17.9)	786 (17.9)
No	578 (7.4)	237 (6.9)	341 (7.8)
Don’t know	5,848 (74.7)	2,573 (75.2)	3,275 (74.7)

Received diptheria, pertussis, and tetanus vaccine, N (%)			

Yes	1,415 (18.1)	626 (18.3)	789 (17.9)
No	541 (6.9)	212 (6.2)	329 (7.5)
Don’t know	5,868 (75.0)	2,584 (75.5)	3,284 (74.6)

Received measles vaccine, N (%)			

Yes	1,561 (20.0)	685 (20.0)	876 (19.9)
No	561 (7.2)	226 (6.6)	335 (7.6)
Don’t know	5,702 (72.9)	2,511 (73.4)	3,191 (72.5)

Received TB vaccine, N (%)			

Yes	54 (0.7)	22 (0.6)	32 (0.7)
No	7,326 (92.5)	3,131 (91.5)	4,105 (93.2)
Don’t know	535 (6.8)	269 (7.9)	266 (6.1)

Received hepatitis B vaccine, N (%)			

Yes	54 (0.69)	22 (0.6)	32 (0.7)
No	7,258 (92.8)	3,141 (91.8)	4,117 (93.5)
Don’t know	513 (6.56)	259 (7.6)	254 (5.8)

Received tetanus vaccine, N (%)			

Yes	1,881 (24.0)	9 (0.3)	1,872 (42.5)
No	5,462 (69.8)	3,154 (92.2)	2,308 (52.4)
Don’t know	482 (6.2)	259 (7.6)	223 (5.1)

Received tetanus booster vaccine, N (%)			

Yes	1,285 (16.4)	298 (8.7)	987 (22.4)
No	6,044 (77.3)	2,863 (83.7)	3,181 (72.3)
Don’t know	495 (6.3)	260 (7.6)	235 (5.3)

Received rabies vaccine, N (%)			

Yes	46 (0.6)	17 (0.5)	29 0.7)
No	7,268 (92.9)	3,138 (91.7)	4,130 (93.8)
Don’t know	511 (6.5)	257 (7.8)	244 (5.5)

Self-report of diseases			

Hypertension, N (%)			

Hypertensive	487 (6.2)	130 (3.8)	357 (8.1)
Normal	7,338 (93.8)	3,292 (96.2)	4,046 (91.9)

Diabetes, N (%)			

Diabetic	102 (1.3)	44 (1.3)	58 (1.3)
Normal	7,723 (98.7)	3,378 (98.7)	4,345 (98.7)

Blood test results			

HIV status, N (%)			

Negative	7,185 (92.4)	3,197 (94.0)	3,988 (91.1)
Positive	593 (7.6)	204 (6.0)	389 (8.9)

Hepatitis B, N (%)			

Negative	7,536 (97.3)	3,268 (96.5)	4,268 (97.9)
Positive	210 (2.7)	117 (3.5)	93 (2.1)

Hepatitis C, N (%)			

Negative	7,536 (97.3)	3,268 (96.5)	4,268 (97.9)
Positive	210 (2.7)	117 (3.5)	93 (2.1)

## Uniqueness of the UGR

The UGR participants are part of the GPC, an active cohort, whose population is well characterized, with GPS coordinates for all households known, and >95% of households agree to participate in studies.^3^ A plethora of longitudinal clinical data also exists for UGR participants (see Table 1), which can be useful in investigating causation of various communicable and non-communicable diseases in a general population setting. Additionally, marked genetic diversity has been reported among UGR samples, where 41.5 million SNPs were called in the sequence data, of which 9.5 million SNPs were novel variants that are not present in the 1000 Genomes project 3 and the African Genome Variation Project reference panels.^4^ The uniqueness of the ethnicities in UGR also serves as an important contribution to the genomic landscape within Africa and globally.

## Contributions of the UGR to scientific discoveries

The UGR data were included in a study that undertook a GWAS in 34 cardiometabolic traits, including lipid, anthropometry traits, blood cell indices, and HbA1c, and reported novel loci associated with anthropometric, hematological, lipid, and glycemic traits among African populations from Uganda, Ghana, Kenya, Nigeria, and South Africa.^4^ This study identified 43 distinct signals statistically associated with at least one trait and, more importantly, established that a p value of 5 × 10^−9^ is more relevant for populations from Africa that have high genetic diversity due to the relatively low levels of linkage disequilibrium (LD) in their genomes.^4^ In another study conducted using the UGR,^9^ we reported the first ever GWAS of kidney function (expressed as estimated glomerular filtration rate; eGFR) in continental Africa. This study validated two loci previously reported at glycine amidinotransferase (*GATM*) and hemoglobin beta (*HBB*) loci that are associated with chronic kidney disease.^9^ By leveraging clinical relatedness and correlations among phenotypes, we have also used the UGR data to explore the power of multivariate GWASs to identify genetic risk factors implicating pleiotropic effects in blood cell traits.^10^^,^^11^ Our results provided a framework for the combination of multiple phenotypes in multivariate GWAS analysis and demonstrated that multivariate genotype-phenotype methods increase power to identify novel genotypes that associate with the phenotype as compared to standard univariate GWASs in the same dataset. We have also used the UGR together with a South African Zulu cohort to conduct the first GWAS of body shape among Africans.^12^ Our results implicated variants in *FGF12*, *GRM8*, *TLX1NB*, and *TRAP1* to be associated with body shape, and we interestingly observed that a SD change in body shape was associated with increases in blood pressure and blood lipids.^12^ Using the UGR and other datasets, we recently were able to show that genetic risk scores derived from data of African American individuals enhance polygenic prediction of lipid traits and type 2 diabetes mellitus (T2DM) in Sub-Sahara Africans, but prediction varied greatly between another dataset from South Africa and our East African genomic data.^13^^,^^14^ We have also demonstrated the Mendelian randomization (MR) evidence of relation between lipid trait and T2DM,^15^ metabolic traits, and stroke.^9^ Collectively, our studies show a need for improved representation of Africans in genomic studies and ensuring the generalization of findings for genomic medicine. This is further supported by findings from another study.^16^ The UGR data have also been used to create a genotype imputation reference panel using UG2G available from the Sanger Imputation Service (https://imputation.sanger.ac.uk).

## Contribution to collaborative studies and future directions

We contribute to global genetic studies through partnerships and consortia, such as the African Partnership for Chronic Disease Research (APCDR), an international network of research groups that collaborate to support and promote collaborative chronic disease research across Africa. An initiative created in response to the changing distribution of communicable diseases and the rising burden of NCDs, as well as the recognition that low- and middle-income countries (LMICs), including those in Sub-Saharan Africa, will need to expand their health care capacities to effectively respond to these epidemiological transitions.

We combine research expertise with three other MRC units (MRC Integrative Epidemiology Unit, MRC Population Health Research Unit, and MRC Unit for Lifelong Health and Aging), and we hope to utilize the UGR data to (1) investigate the potential to use MR to assess the generalizability of existing drugs (e.g., statins, anti-diabetics, and anti-hypertensives); (2) identify the potential to tailor drugs with pilot studies focusing on established pharmacological targets to specific subpopulations (e.g., *CETP*, *HMGCR*); and (3) see how changes in genetic architecture affect efficacy estimates in different groups.

The UGR contributes to the CARDINAL (CARDiometabolic Disorders IN African-ancestry PopuLations) consortium, which is a study site of an NIH-funded Polygenic Risk Methods in Diverse Populations (PRIMED) Consortium (https://primedconsortium.org/).The CARDINAL^17^ aims to integrate phenotype and genomic datasets from 50,000 African individuals from seven cohort studies and evaluate polygenic risk scores (PRSs) to develop a novel method that considers ancestry-specific genomic regions to improve PRS prediction in populations with genetic substructure.

Furthermore, the UGR data have also been recently included into the Meta-Analyses of Glucose and Insulin-related Variables Consortium (MAGIC). The MAGIC study seeks to identify additional loci that influence glycemic and metabolic traits.^18^ The UGR also contributes to the International HundredK + Cohorts Consortium (IHCC), which aim is to create a global platform for translational research.^19^

The UGR presents opportunity to contribute key phenotypes, such as lipids, blood cell traits, kidney functions, etc., to other consortia. The GBMI is a great platform where most of the phenotypic data described in Table 1 can contribute to global meta-analysis with an opportunity to measure not previously collected phenotype using resources at the MRC/UVRI and LSHTM as described in Figure 2. We believe that team science allows scientists to make the most progress toward breakthrough discoveries that benefit human health.

The GPC comprises more than 22,000 participants, and being a live cohort creates opportunity for genotyping DNA samples from more GPC participants to add to the UGR. We also hope to sequence more samples at higher coverage in order to increase the genetic diversity of the UGR, which could lead to identification of more novel and private alleles and ultimately contribute to fine-mapping of alleles that could be associated with several different diseases and traits. Higher coverage will also provide a reference panel with increased genome coverage, which could improve imputation capacity.

Since participants in the UGR can be traced and involved in future studies, there is opportunity to collect fresh samples like blood, urine, stool, and saliva. The opportunity for availability of these samples can be utilized to design proteomics, metabolomics, single-cell genomics, and other omics studies in the UGR to understand their contribution to disease and traits.

## Conclusions

The UGR is designed to directly impact biomedical and genetic research of health and disease in Uganda, Africa, and globally. The UGR has become one of the model genomic resources in Africa and offers training opportunities to researchers from Uganda and the world at large. Here, we present an overview of the UGR, showcase its broad range of phenotypic data, and highlight the genetic discoveries from UGR to date. In the next few years, the UGR will continue to grow in sample size and will include proteomics, metabolomics, and single-cell genomic studies.

## Limitations of the UGR

The UGR comprises participants who are predominantly of Bagandan ethnicity (>75%) and thus may not be representative of the entire Ugandan population. There is a need to include Ugandan participants of other ethnicities to improve the generalizability findings from the UGR.

The whole-genome sequence data of the UGR were sequenced at a lower coverage (4×), and thus, some novel variants may have been missed. There is a need for sequencing of the UGR data at higher coverage in order to include more variants, some of which may be novel and could be of importance to different conditions or diseases.

## Data access and sharing of the UGR data

Request for resources and information should be directed to UGR’s Data Access Committee (via the email: UGR@mrcuganda.org). The UGR’s individual-level data, genotype, and sequence data are available under managed access to researchers. Requests for access will be granted for all research consistent with the consent provided by participants. This would include any research in the context of health and disease that does not involve identifying the participants in any way.

The array and low- and high-depth sequence data have been deposited at the European Genome-phenome Archive (EGA, https://www.ebi.ac.uk/ega/, accession numbers EGAS00001001558/EGAD00010000965, EGAS00001000545/EGAD00001001639, and EGAS00001000545/EGAD00001005346, respectively. Requests for access to data may be directed to UGR@mrcuganda.org. Applications are reviewed by data access committee (DAC), and access is granted if the request is consistent with the consent provided by participants. The data producers may be consulted by the DAC to evaluate potential ethical conflicts. Requestors also sign an agreement that governs the terms on which access to data is granted.

However, full GWAS summary statistics of UGR is freely available on GWAS catalog https://www.ebi.ac.uk/gwas/ with study accession numbers: GCST009041 (eosinophil counts), GCST009042 (total cholesterol levels), GCST009043 (LDL cholesterol levels), GCST009044 (HDL cholesterol levels), GCST009045 (triglyceride levels), GCST009046 (aspartate aminotransferase levels), GCST009047 (alanine aminotransferase levels), GCST009048 (serum albumin levels), GCST009049 (serum alkaline phosphatase levels), GCST009050 (gamma glutamyl transferase levels), GCST009051 (bilirubin levels), GCST009052 (diastolic blood pressure) GCST009053 (systolic blood pressure), GCST009054 (hemoglobin A1c levels), GCST009055 (height), GCST009056 (weight), GCST009057 (body mass index), GCST009058 (waist circumference), GCST009059 (hip circumference), GCST009060 (waist-hip ratio), GCST009061 (white blood cell count), GCST009062 (red blood cell count), GCST009063 (mean corpuscular hemoglobin), GCST009064 (mean corpuscular hemoglobin concentration), GCST009065 (mean corpuscular volume), GCST009032 (red blood cell distribution width), GCST009033 (hematocrit), GCST009034 (hemoglobin measurement), GCST009035 (mean platelet volume), GCST009036 (platelet count), GCST009037 (lymphocyte count), GCST009038 (monocyte count), GCST009039 (basophil count), GCST009040 (neutrophil count).
